# Identification of a Red Pigment-Producing Strain of *Arthrobacter* spp. and the Stability of Its Pigments

**DOI:** 10.3390/microorganisms13092003

**Published:** 2025-08-27

**Authors:** Jinjun Wang, Mingliang Yang, Xinru Gao

**Affiliations:** 1 Key Laboratory of Arable Land Quality Monitoring and Evaluation, Ministry of Agriculture and Rural Affairs, Yangzhou University, Yangzhou 225009, China; 2 College of Environmental Science and Engineering, Yangzhou University, Yangzhou 225127, China; yasmine0787@163.com (M.Y.); gxr0207@163.com (X.G.)

**Keywords:** red pigment, *Arthrobacter* spp., fermentation conditions, stability

## Abstract

With the rise of environmental protection and health topics in recent years, microbial production of red pigments has gradually become a research hotspot. Red pigment possesses biological properties such as anticancer and antioxidant activities and has a wide range of potential applications in the fields of food and medicine. In this paper, a red pigment-producing strain was screened from rice soil to provide a reserve for obtaining natural and safe red pigments. Methods: The strain LSY1-2 was identified using morphological and 16S rDNA molecular biological identification. The fermentation conditions for red pigment production were optimised to improve pigment yield, and the best conditions were analysed using response surface methodology. Finally, the stabilisation conditions of red pigment were analysed to determine the difficulty of retention. Results: The molecular ecology was identified as the bacterium *Arthrobacter* sp. of the genus *Arthrobacter*. The optimal red pigment production medium for the strain was determined by a one-way test with the carbon source beef extract, the nitrogen source peptone, the inoculum size 2%, the temperature 27 °C, the pH value 7, and the rotational speed 160 rpm. Response surface optimisation determined the optimal red pigment production conditions as the incubation temperature of 26.43 °C, the pH value of 6.89, and the rotational speed of 162.77 rpm, which resulted in the yield of red pigment under these optimal conditions as 0.883 U/mL. The stability of red pigment was best under the condition without light, and poorer under conditions of above 50 °C, strong acid, strong alkali, and more than 3% oxidant, and Fe^3+^ had a greater effect on the stability. Conclusions: Strain LSY-1 can produce stable red pigment under the optimised red pigment-producing conditions, which provides a reference for the large-scale production of natural red pigment and subsequent related research.

## 1. Introduction

Pigments, also known as colouring agents, are used to impart certain colours to substances by selectively absorbing specific wavelengths of light and reflecting other wavelengths [1]. Compared with synthetic pigments, which are difficult to degrade and toxic, natural pigments have significant environmental advantages, and microbial-derived natural pigments have attracted much attention due to their stable production and low cost [2,3,4,5]. Microorganisms with the ability to secrete pigments are diverse, and the natural pigments they produce are also diverse [6]. As a potential pool of pigment resources, the development and research around microbial pigments are of great significance.

While most of the current sources of pigments are plant and animal pigments, microbial pigments are usually not affected by raw materials and seasons, and are therefore considered as an important source for industrial production applications [7]. As a class of secondary metabolites, despite the advantages of the exploitation of microbial pigments, there are still some limitations that need to be overcome in terms of large-scale production, such as lower yields and inconsistent product quality [8,9], as well as the inability to produce stable, non-toxic red pigments [10,11]. Therefore, it is necessary to develop more efficient microbial pigment production methods to solve the problems faced. At the present stage, red-producing microorganisms are mainly improved through the use of genetic engineering techniques, using physicochemical factors to mutate strains [12], or by changing and modifying strain genes to control and improve the synthesis of pigment production [13], and by optimising the culture and fermentation conditions to enhance the fermentation capacity of strains, so as to obtain strains with higher production of pigments [14].

In Wang Jinjun’s laboratory at Yangzhou University, we studied a red pigment-secreting strain isolated from rice field soil and named it LSY1-2. By identifying the strain morphologically and molecularly, as well as from optimising the fermentation conditions of the strain for red pigment production, the stability of red pigment, and the purification and characterisation of the pigment, preliminary experimental conclusions were drawn for the subsequent research on the bioactivity of red pigment as well as its industrial development and utilisation.

## 2. Materials and Methods

### 2.1. Materials

#### 2.1.1. Sample Sources

A red pigment-producing strain was found among the bacteria isolated from the soil of rice fields.

#### 2.1.2. Culture Medium

Nutrient broth medium (NB): 10 g of peptone, 3 g of beef paste, 5 g of sodium chloride, 1000 mL of distilled water, pH = 7.0~7.4; sterilised at 121 °C for 20 min.

NB solid medium: 10 g of peptone, 3 g of beef paste, 5 g of sodium chloride, 18 g of purified agar powder, 1000 mL of distilled water, pH = 7.0~7.4; sterilised at 121 °C for 20 min.

#### 2.1.3. Data Analysis

Bar charts were created using Origin 2024 software (OriginLab Corporation, Northampton, MA, USA), and data were analysed for significance using IBM SPSS Statistics (version 29.0, IBM Corporation, Armonk, NY, USA).

### 2.2. Methods

#### 2.2.1. Morphological Identification and Molecular Biological Identification of Strains

Genomic DNA of the strains was extracted with the Bacterial Genomic DNA Extraction Kit (Transgen Bio, Beijing, China), and 16S rDNA sequence analysis was performed [15]. The PCR products [16] were sent to Shanghai Sangong Biological Company for sequencing, and then BLAST+ (version 2.16.0+, New York, NY, USA) analysis was carried out by NCBI to determine the species relationship of the strains. The sequences with higher homology were selected, and the phylogenetic tree was constructed using MEGA11.

#### 2.2.2. Determination of the Relative Optimal Growth Temperature and Pigment Solubility of the Strain

Five temperatures of 16 °C, 21 °C, 26 °C, 31 °C, and 36 °C were set. Each temperature gradient was set three times in parallel. They were cultivated at constant temperature for 36 h, and the growth of the strains at each temperature was observed and compared.

The seed solution was incubated at 26 °C and 180 rpm for 24 h. After centrifuging at 4000 rpm for 10 min, the supernatant was removed, resuspended in solvent, and extracted for 15 min. It was centrifuged again to obtain the red pigment extraction solution. The red pigment was extracted using four organic solvents and one inorganic solvent, and the one with the best results was selected.

#### 2.2.3. Effects of Different Single-Factor Conditions on Red Pigment Production

- Effects of different carbon sources, nitrogen sources, and inoculum amount of culture medium on pigment yield:
- Beef extract, glucose, sucrose, glycerol, and ethanol were selected as the carbon sources of the screened medium, respectively, and the rest of the components were the same. The inoculum was 2%, and the medium was incubated at 27 °C and 160 rpm for 48 h. Each temperature gradient was repeated three times. The Optical Density (OD) value was measured, and the colour value of red pigment was calculated to compare the effect of each carbon source on the red pigment yield.
  Peptone, yeast extract, (NH_4_)_2_SO_4_, and NH_4_Cl were selected as the nitrogen source of the screened medium, and the rest of the components were the same. The same method was used as in the carbon source experiment.
  The selected carbon sources and nitrogen sources were used as the medium components, and the rest of the components were unchanged. Six different strains of 1%, 2%, 3%, 4%, 5%, and 6% were selected as inoculum and cultured under the same conditions, respectively. Each temperature gradient was repeated three times. The OD value was measured, the colour value of red pigment was calculated, and the inoculum quantity was selected.
- Effects of different pH, temperature, and rotational speed on pigment yield:
  The selected carbon and nitrogen sources were used as the medium components, while the rest of the components remained unchanged. The pH of the medium was adjusted to 5, 6, 7, 8, and 9, respectively, and cultured under the same conditions with the optimal amount of bacteria. Each temperature gradient was repeated three times. The OD value was measured, the red pigment colour value was calculated, and the optimal pH value was selected for comparison.
  The temperature was set at 21 °C, 24 °C, 27 °C, 30 °C, and 33 °C, and the operation was the same as above.
  The rotational speed was set at 100 rpm, 130 rpm, 160 rpm, 190 rpm, and 220 rpm, and the operation was as above.

#### 2.2.4. Optimisation of Response Surface Conditions

Response surface methodology (RSM) typically uses single-factor experimental results as a basis for designing multivariate equations to analyse the effects of multiple factors and their interactions on response values. Based on the results of the single-factor experiment, three factors that have a significant impact on the colour value of red pigments were selected: temperature, rotation speed, and pH value. Colony growth is most vigorous at 27 °C, 160 rpm, and pH = 7.0. The range was set with small adjustments around these three values. The Box–Behnken test was designed using Design Expert 8.0.6 (2010, America) to further optimise the red pigment production. Regression analysis was performed to obtain the regression model, and a significance test was performed to generate the quadratic polynomial regression equation. Table 1 presents the ranges of all tested factors. Using the control variable method, it was ensured that only one variable was changed in each experiment, while keeping all other conditions constant. The experiment was conducted with temperature, rotation speed, and pH as variables. The strain was fermented in NB medium for 48 h to obtain the fermentation broth. The method for extracting red pigment was the same as that described in Section 2.2.2. The absorbance (OD) was measured and the colour value was calculated. Colour value (U/mL) = OD × dilution factor.

#### 2.2.5. Effect of Light, Temperature, pH, Oxidants and Metal Ions on the Stability of Red Pigments

Five amounts of 15 mL of equal concentrations of red pigment methanol solution (hereinafter referred to as pigment solution) were aspirated in a clean test tube and placed under five different light environments, i.e., UV light, outdoor sunlight, indoor sheltered light, indoor natural light, and indoor brown bottles. Samples were taken hourly to determine the absorbance of the red pigment solution at the maximum absorption wavelength.

Equal amounts of pigment solutions were placed in test tubes wrapped in tin foil at constant temperatures of 4 °C, 25 °C, 30 °C, 40 °C, 50 °C, and 60 °C. Absorbance of the solutions was measured at 0, 0.5, 1, 3, 5, and 7 h.

Eight 10 mL portions of the pigment solution were pipetted, and the pH values were adjusted to 2, 4, 6, 7, 8, 9, 10, and 13 with 0.1 mol/L HCl and 0.1 mol/L NaOH. The solution was shaken well and left to stand away from light. The absorbance was measured at 0 h, 0.5 h, 1 h, 3 h, 7 h, and 24 h, respectively.

An amount of 15 mL of each pigment solution was pipetted into a 25 mL volumetric flask, respectively. A 30% hydrogen peroxide (H_2_O_2_) solution was added in the ratio of 0%, 1%, 2%, 3%, 4%, and 5%, and the solution was fixed with methanol solution. It was placed in a water bath at 25 °C, protected from light. The absorbance of the pigment solution was measured after 0 h, 1 h, 2 h, 3 h, and 24 h. The absorbance of the pigment solution was measured at 0 h, 1 h, 2 h, 3 h, and 24 h.

## 3. Results

### 3.1. Morphological Characteristics and Molecular Biological Identification of Red Pigment-Producing Strains

**Figure 1:**
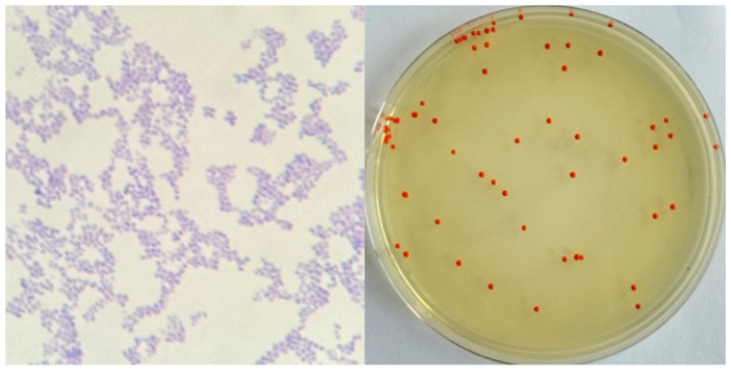
Morphological observation of the strain.

As shown in Figure 1, after 48 h of culture, the strains grew well and formed red round single colonies, with obvious colony characteristics of the genus *Arthrobacter* [17]. The strain was purple after Gram staining and was Gram-positive.

**Figure 2:**
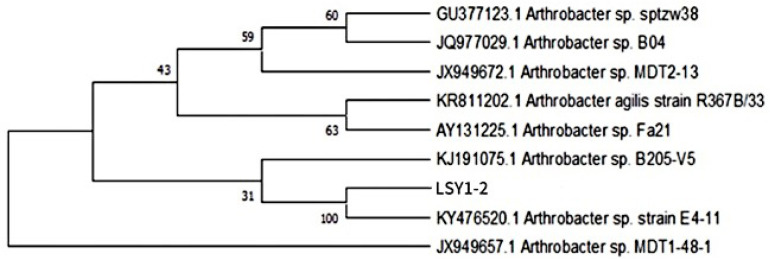
Phylogenetic tree of the strain LSY1-2.

The sequencing results were compared with the NCBI database for blast comparison, and the strains with high similarity to the target strains were selected; the phylogenetic tree was constructed by MEGA 7.0 software, and the results are shown in Figure 2. The homologous similarity between the strains and species of the genus *Arthrobacter* was greater than 99%. Therefore, strain LSY1-2 was initially identified as the cyanobacterial fungus *Arthrobacter* sp. LSY1-2.

### 3.2. Determination of the Relative Optimum Growth Temperature and Pigment Solubility of Red Pigment-Producing Strains

**Figure 3:**
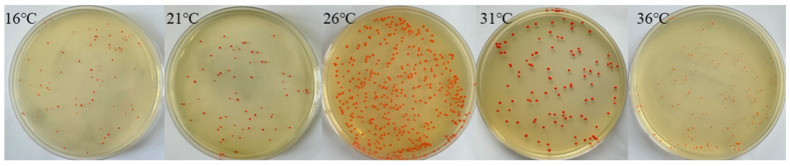
Strain community growth at different temperatures.

As shown in Figure 3, different temperatures had a large effect on the growth of the strains. When the temperature range was between 16 °C and 36 °C, the best growth condition was at 26 °C. The quantity was the highest and the colour was the darkest. Therefore, the temperature of the subsequent experiments was uniformly set at 26 °C.

**Figure 4:**
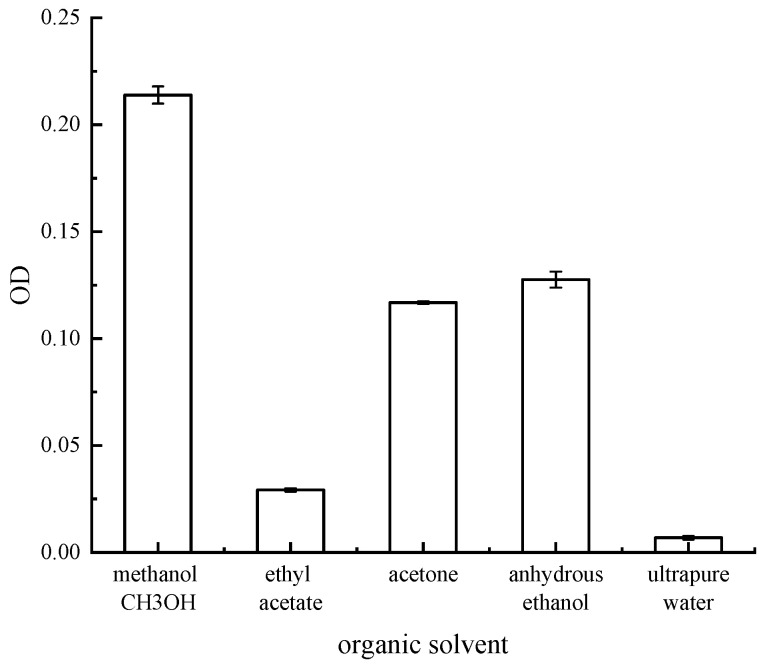
OD value of different organic solvent pigment solutions.

As shown in Figure 4, the solubility of red pigment produced by the strain varied in different organic solvents. Among the five different organic solvents, methanol showed the best solubility for red pigment. Therefore, all the pigment extraction solutions in the later section were red pigment methanol solutions.

### 3.3. Effect of Single-Factor Conditions on Pigment Yield

#### 3.3.1. Effects of Medium Carbon Source, Nitrogen Source, and the Amount of Bacteria Received on Pigment Yield

**Figure 5:**
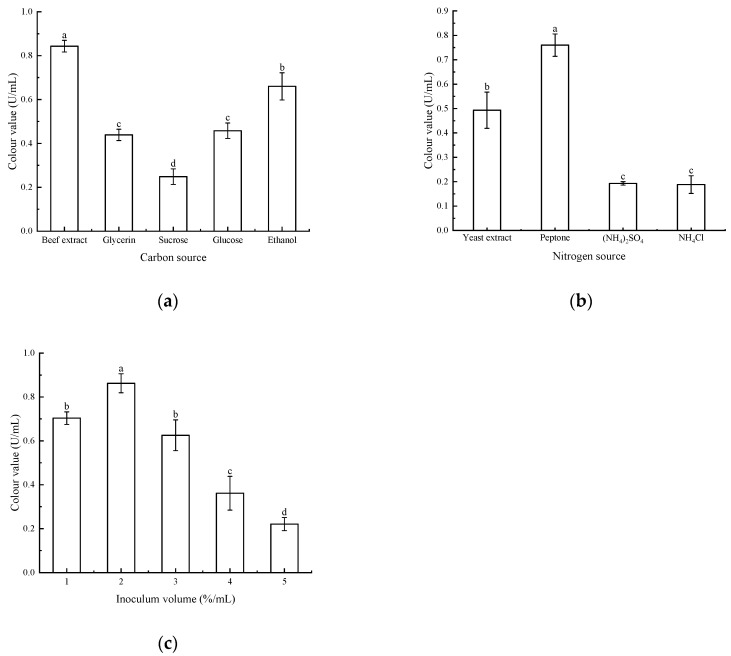
(**a**) Effect of different carbon sources on pigment colour values; (**b**) effect of different nitrogen sources on pigment colour values; (**c**) effect of different inoculum amounts on pigment colour values. Note: Different letters indicate significant differences between the two groups (*p* < 0.05).

As shown in Figure 5, different carbon sources, nitrogen sources, and the amount of bacteria received had a large effect on the pigment yield of the strains. Using beef extract and peptone as carbon and nitrogen sources for red pigment fermentation, an inoculation level of 2% yielded the highest colour value and maximum red pigment yield. This showed significant differences compared to other culture conditions and inoculation levels. Therefore, the best pigment yield was obtained by using beef extract and peptone as carbon and nitrogen sources for the fermentation culture of strain *Arthrobacter* sp. LSY1-2 at an inoculum of 2%.

#### 3.3.2. Effect of pH, Temperature, and Rotational Speed on Pigment Production

**Figure 6:**
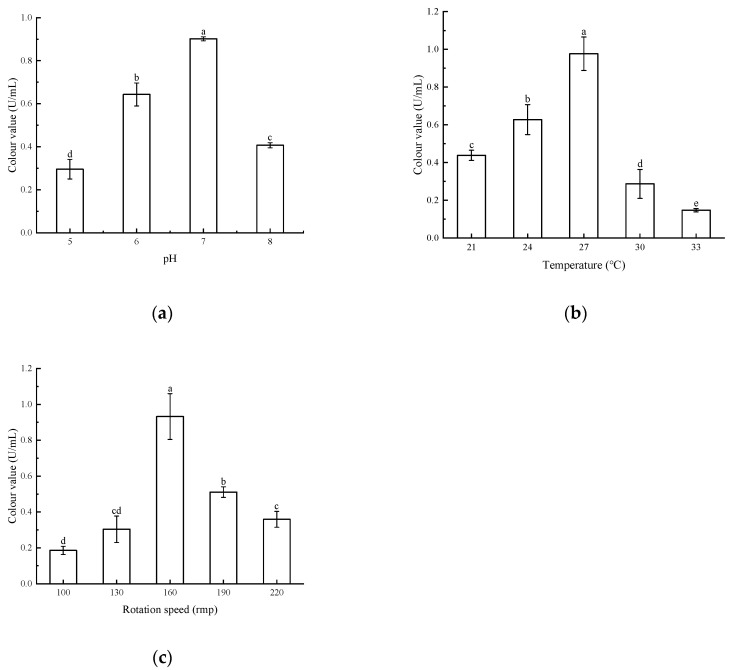
(**a**) Effect of different pH on pigment colour values; (**b**) effect of different temperatures on pigment colour values; (**c**) effect of different rotational speeds on pigment colour values. Note: Different letters indicate significant differences between the two groups (*p* < 0.05).

The results are shown in Figure 6. The highest pigment colour value of the strains was observed at pH 7, temperature 27 °C, and rotational speed 160 rpm. This difference was significant compared to the other conditions. Decreasing or increasing pH, too high or too low a temperature, and too fast or too slow a rotational speed will lead to the decrease of colour value of the pigment. Therefore, the strain *Arthrobacter* sp. LSY1-2 was more likely to produce large amounts of pigment when it was at medium pH = 7, 27 °C, and 160 rpm.

### 3.4. Response Surface Test Optimisation Results

#### 3.4.1. Model Establishment and Significance Analysis

Based on the results of the one-factor experiment, the three factors that had the greatest influence on the pigment colour value produced by strain *Arthrobacter* sp. LSY1-2 were selected: A (temperature 24 °C, 27 °C, and 30 °C), B (pH 6, 7, and 8), and C (rotational speed of 130 rpm, 160 rpm, and 190 rpm) to design the Box–Behnken test for response surface optimisation using red pigment colour value as the response value. The optimal conditions for expanding the yield of pigment secreted by the strain were explored. The results of Box–Behnken test are presented in Table 2.

The regression equation was obtained by multivariate fitting using Design Expert (Version 8.0.6).

Y (colour value) = −27.8958 + 1.1005A + 2.2758B + 0.0786C − 0.0029AB + 0.0003AC +  0.0004BC − 0.0212A^2^ − 0.1646 B^2^ − 0.0002C^2^ (1)

A, B, and C are the actual values of the tested factors.

The analysis of variance (ANOVA) and significance calculation of the above equation were carried out, and the results are presented in Table 3. From Table 3, it is clear that the model is highly significant, as indicated by the *p*-value < 0.01. The misfit term having a *p*-value > 0.05 is not significant, and the model fits the data well with a high degree of confidence. A C.V.% < 10% represents high experimental confidence and precision, while the effective signal to noise ratio is 28.623, >4, indicating a reasonable degree of model precision. The multivariate correlation coefficient R^2^ = 0.9925. In conclusion, it shows that the regression model has a good degree of regression and good adaptability, and can be used to predict the optimal yield conditions for pigment colour values using this equation.

Moreover, ANOVA also showed that the effect of each factor on pigment colour value was A > C > B, i.e., temperature > speed > pH. The primary terms A, B, and C had highly significant effects on the results (*p* < 0.01), while the interaction terms AB, AC, and BC had insignificant effects on the results (*p* > 0.05), indicating that the effects of the three on the colour value of the pigment were not significant. Meanwhile the secondary terms A^2^, B^2^, and C^2^ had highly significant effects on the results (*p* < 0.001).

#### 3.4.2. Response Surface Analysis

**Figure 7:**
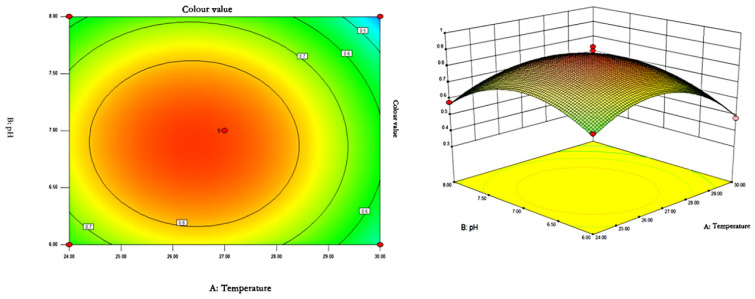
Contour plots and response surface plots of the interactive effects of temperature and pH on pigment colour values.

**Figure 8:**
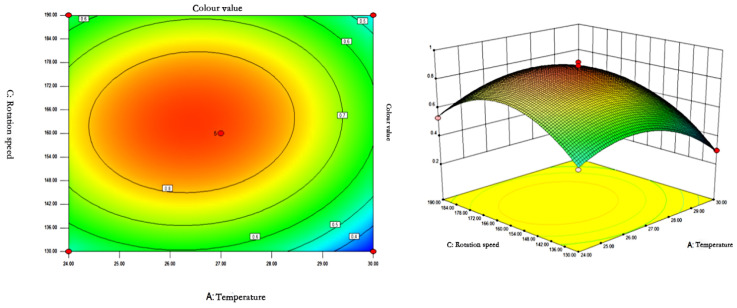
Contour plots and response surface plots of the interactive effects of temperature and rotational speed on pigment colour values.

**Figure 9:**
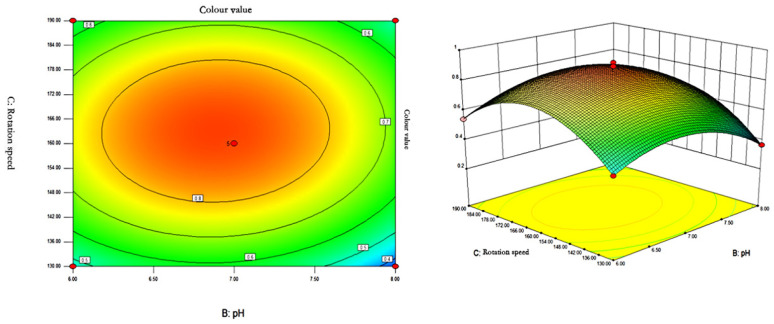
Contour plots and response surface plots of the interactive effects of pH and rotational speed on pigment colour values.

The response surface curves and contours of the effects of temperature, pH, and rotational speed on the colour value of red pigments are shown in Figure 7, Figure 8 and Figure 9.

As can be seen from Figure 7, Figure 8 and Figure 9, the shape of the contour plots of the effect of temperature and pH on the colour value of pigments tends to be close to a circle, indicating that the interaction of the two factors does not have a significant effect on the colour value of red pigments. The rotational speed is constant, and the colour value tends to increase and then decrease with the increase of temperature and pH. The colour value of the pigment could reach a higher value when the temperature was between 26.37 and 26.48 °C and the pH was between 6.85 and 6.89.

The contours of the interaction of temperature and rotational speed on colour value were a closed ellipse, indicating that the interaction had a significant effect on the colour value of the pigment. pH was constant, and the colour value of the pigment showed a tendency to increase and then decrease with the increase of rotational speed and temperature. The colour value of the pigment could reach the maximum at 26.37–26.48 °C and 162.2–163.5 rpm.

The contour lines of the effect of the interaction of pH and rotational speed on the colour value are in the shape of a closed ellipse, indicating that the interaction has a significant effect on the colour value of the pigments. At constant temperature, the colour value of pigment showed a tendency of increasing and then decreasing with the increase of rotational speed and pH. When pH = 6.85–6.89 and rotational speed is 162.2–163.5 rpm, the colour value of pigment can reach the maximum.

Comprehensive analysis using Design Expert 8.0.6 software showed that the optimal fermentation conditions for pigment production by strain *Arthrobacter* sp. LSY1-2 were temperature 26.43 °C, pH 6.89, and rotational speed 162.77 rpm, and the predicted colour value of the pigment produced by the strain was 0.899161 U/mL. In order to validate the reliability of response surface optimisation experiments, the results were verified on the basis of the derived conditions. Due to the influence of the actual conditions, the optimised conditions were modified to 26.5 °C, pH 6.9, and 163 rpm. Three parallel experiments were set up, and the mean value of the results was 0.883 U/mL, which was less different from the predicted value. Therefore, the Box–Behnken response surface optimisation method is feasible for the optimisation of the pigment-producing ability of strain LSY-1 and has some practical application value.

### 3.5. Effect of Light, Temperature, pH, Oxidants, and Metal Ions on the Stability of Red Pigments

**Figure 10:**
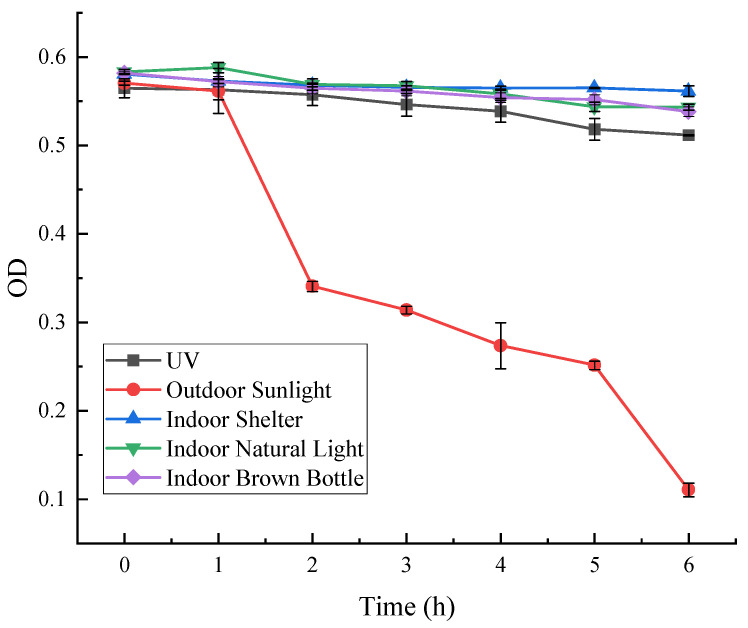
Effect of light on the stability of red pigments.

As can be seen from Figure 10, the absorbance of red pigment irradiated by outdoor sunlight at OD = 490, decreased significantly with time and was less stable. After 2 h of irradiation, the red pigment preservation decreased to 60%, and after 6 h, only about 18% of the preservation remained. The stability of red pigment under the rest of the light conditions was better, and the pigment decomposition rate was lower. Under the light-avoidance condition, the retention rate after 6 h was 97%.

**Figure 11:**
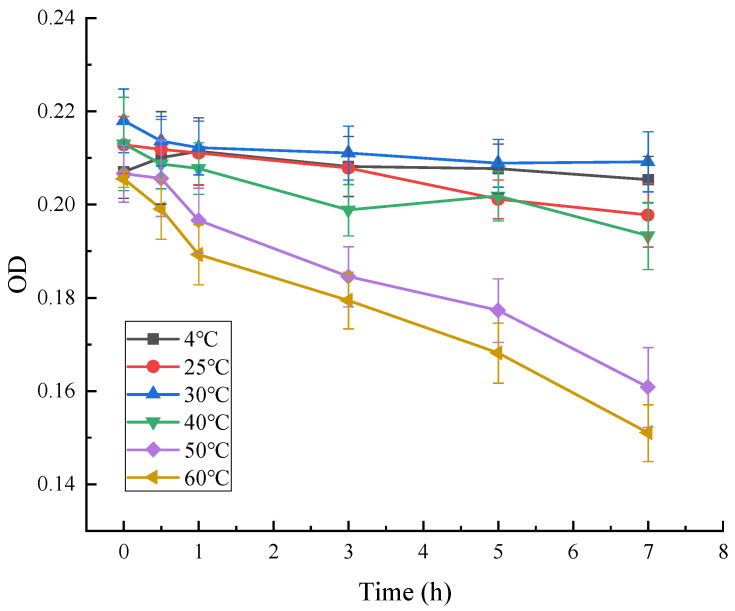
Effect of temperature on the stability of red pigments.

As can be seen from Figure 11, the absorbance of red pigment at OD = 490 decreased significantly with time at different temperatures, and the residual percentage of red pigment at 4 °C, 25 °C, 30 °C, and 40 °C decreased to about 95%, 90%, 93%, and 91%, respectively, after 7 h. The residual rate of red pigment at 50 °C and 60 °C decreased to about 93% after 1 h, and to about 74% and 75% after 7 h. The results showed that the red pigment produced by the strain decreased significantly after 7 h at 4 °C, 25 °C, 30 °C, and 40 °C, respectively. The results showed that the red pigment produced by the strain was stable in the range of 4–40 °C, while at 50 °C and above, the red pigment showed high temperature sensitivity and the pigment properties would change. It was concluded that the red pigment produced by *Arthrobacter* sp. LSY1-2 was not resistant to high temperature, and was stable at low or normal temperature for a short period of time.

**Figure 12:**
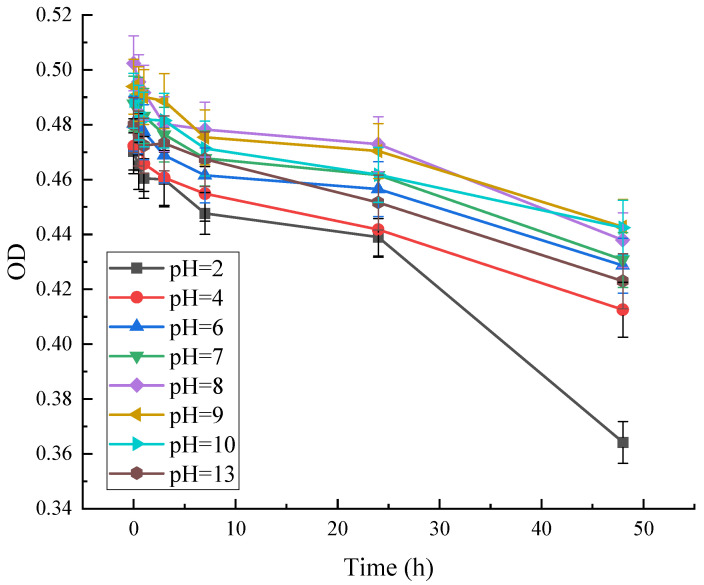
Effect of pH on the stability of red pigments.

As can be seen in Figure 12, the absorbance of red pigment at OD = 490 decreased with time at different pH. At pH = 2, the OD was at its lowest, and the pigment residue decreased to 77%. This indicates that the stability of the pigment is poor under strong acid conditions. The variation of red pigment at other pH conditions was less. It shows that the red pigment has better stability under weak acid, neutral, and weak base conditions.

**Figure 13:**
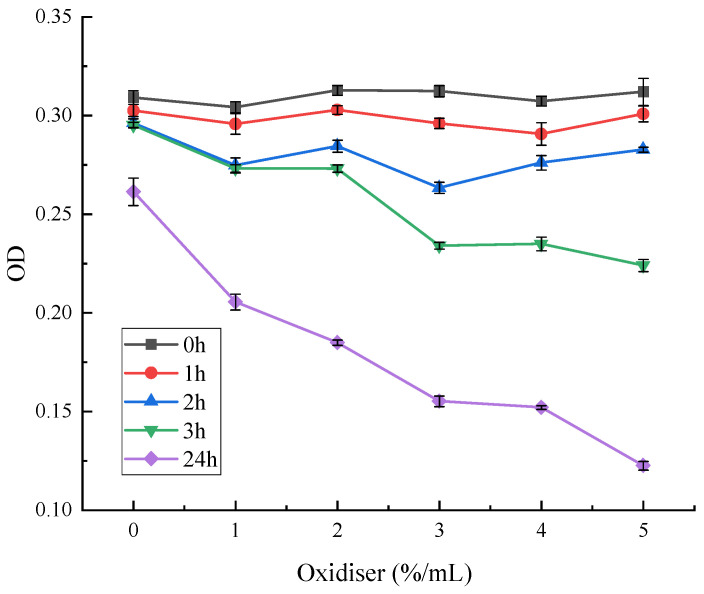
Effect of oxidising agents on the stability of red pigments.

As can be seen from Figure 13, different concentrations of oxidising agents had less effect on red pigment stability during the treatment time 0–2 h. The red pigment stability was significantly reduced after three hours. Furthermore, the higher the concentration of hydrogen peroxide, the lower the residual amount of red pigment. At a concentration of 3%, the red pigment OD value was significantly reduced. At a concentration of 5%, the retention decreased to 77%. Then, 24 h later, the OD values of the methanol solutions of red pigment were significantly reduced at all concentrations. The pigment residue was only about 46% under hydrogen peroxide treatment at a concentration of 5%.

**Figure 14:**
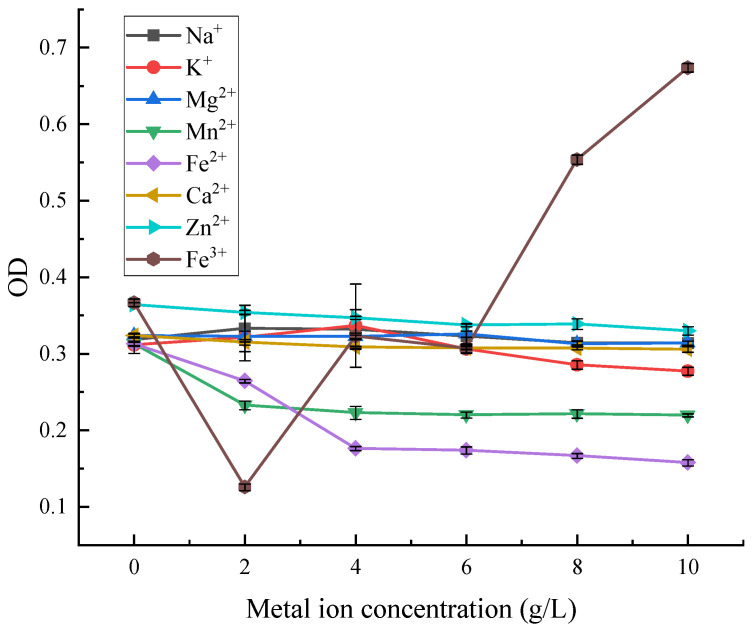
Effect of metal ions on the stability of red pigments.

As can be seen from Figure 14, among the eight metal ions with different concentrations, Mn^2+^ decreased the OD value of red pigment, and then it gradually levelled off. Fe^2+^ and Fe^3+^ had obvious achromatic effects on red pigment, and Fe^3+^ was the most obvious. Fe^2+^ had a stronger achromatic effect on red pigment in the range of 0–4 gL^−1^, with the loss of red pigment of about 45%. The effect of Fe^3+^ on red pigment stability was the greatest in the range of 0–4 gL^−1^, where the loss of red pigment was almost 99% at a particular concentration. With the increase of Fe^3+^ concentration, the absorbance value of the red pigment solution increased, as the trivalent iron ions in the solution affected the absorbance.

## 4. Discussion

Plant pigments are more environmentally demanding and less stable [18], and animal pigments are less available and more costly [19], and both of them also have the disadvantage of greater difficulty of extraction, so microorganisms have become an important source for obtaining natural pigments [20]. Microbially produced red pigments are highly regarded for their safety and environmental friendliness compared to synthetic pigments [13,21,22]. However, the stability of microbial-derived red pigments is weak [23,24], so it is important to analyse the factors and conditions affecting the stability of microbial-derived red pigments to provide some guarantee for the future large-scale production and application of microbial-derived red pigments [25].

In this study, a red pigment-producing strain from a rice field soil isolate was identified. The location of the root zone is a major factor influencing the diversity and composition of the root-associated bacterial community in rice, and this paper examines strains that, according to location, were found to have potentially different roles [26]. Based on molecular biology results, it was determined that the strain had 99% homology with *Arthrobacter* spp. and could be named *Arthrobacter* sp. LSY1-2. It was identified as an *Arthrobacter* sp., and the optimal culture and fermentation conditions were clarified, which laid the foundation for subsequent large-scale production.

Based on the response surface methodology, the three factors that had the greatest influence on the colour value of the red pigment produced by the strain *Arthrobacter* sp. LSY1-2 were selected for optimisation: pH, temperature, and rotational speed. According to the optimisation protocol based on the validated model, the optimal red pigment colour value produced by the strain was 0.899 U/mL. This value could be achieved under the following fermentation conditions: 26.43 °C, pH 6.89, and a rotational speed of 162.77 rpm. Compared with the traditional natural pigments of plant and animal origin, the fermentation production of this strain is not restricted by seasons and regions, and it is easy to obtain raw materials by using beef extract and peptone as the sources of carbon and nitrogen, which is conducive to the reduction of cost and the realisation of industrial production.

The properties of red pigments were analysed to investigate the solubility of red pigments in different solvents and the effects of temperature, pH, light, oxidants, and metal ions on the stability of red pigments. The stability performance in different solvents, temperature, acid–base, and oxidising environments provides guidance for its possible applications in food, cosmetics, and pharmaceuticals [27].

At this stage, synthetic pigments are still the main choice for people by virtue of their significant advantages of cheapness and stability, but due to their non-negligible hazards to the ecological environment and even to the human body, there is a need to seek more environmentally friendly natural pigments [28]. There is a rich variety of secondary metabolites of microorganisms, and most microbial pigments have certain biological activities, such as antioxidant, antibacterial, and anti-inflammatory properties [29,30]. However, the secretion process is also accompanied by the production of toxic substances [31]. For example, the mycotoxin orange penicillin is also produced during the fermentation of red pigment production by *Aspergillus erythropolis*, which has a certain carcinogenicity rate and can cause adverse effects on human health [32,33], while pigments from certain pathogenic bacteria can also promote bacterial pathogenicity [33]. Therefore, the bioactivity of red pigments produced by bacterial strains needs to be further investigated. For example, the next experiment could explore whether the red pigment produced in this experiment is toxic and what its effects are [20,34,35]. Compared to red pigments synthesised by other methods, what are the differences between red pigments produced by microorganisms and those synthesised by other methods [10,11]? Its application value in multiple industries could be explored. It could then replace synthetic pigments and be widely used in the chemical and pharmaceutical industries.

## Figures and Tables

**Table 1:** Experimental range of each tested factor.

	Factor	Level		
A	Temperature/°C	24	27	30
B	pH	6	7	8
C	Rotation speed/rpm	130	160	190

**Table 2:** Box–Behnken design of experimental program and results.

Serial Number	Temperature (°C)	pH	Rotation Speed (rpm)	Colour Value (U/mL)
1	24	6	160	0.642
2	30	6	160	0.481
3	24	8	160	0.579
4	30	8	160	0.383
5	24	7	130	0.474
6	30	7	130	0.299
7	24	7	190	0.532
8	30	7	190	0.456
9	27	6	130	0.468
10	27	8	130	0.367
11	27	6	190	0.542
12	27	8	190	0.492
13	27	7	160	0.857
14	27	7	160	0.872
15	27	7	160	0.919
16	27	7	160	0.895
17	27	7	160	0.844

**Table 3:** ANOVA results.

Source	Sum of Squares	df	Mean Square	F-Value	*p*-Value Prob > F	Significance
Modelling Model	0.66	9	0.074	102.38	<0.0001	significant
A Temperature	0.046	1	0.046	64.05	<0.0001	
B pH	0.012	1	0.012	16.87	0.0045	
C Rotation speed	0.021	1	0.021	29.7	0.001	
AB	0.0003062	1	0.0003062	0.42	0.5355	
AC	0.00245	1	0.00245	3.4	0.1079	
BC	0.0006502	1	0.0006502	0.9	0.374	
A^2^	0.15	1	0.15	214.19	<0.0001	
B^2^	0.11	1	0.11	158.07	<0.0001	
C^2^	0.25	1	0.25	351.96	<0.0001	
Residual	0.00505	7	0.0007215			
Lack of Fit	0.001449	3	0.000483	0.54	0.6819	not significant
Pure Error	0.003601	4	0.0009003			
Cor Total	0.67	16				
R^2^ = 0.9925		R^2^_adj_ = 0.9828		R^2^_pred_ = 0.9570	C.V.% = 4.52	signal-to-noise ratio = 28.623

Note: 0.01 < *p* < 0.05 means the difference is significant; *p* < 0.01 means the difference is highly significant; and *p* > 0.05 means the difference is not significant.

## Data Availability

The original contributions presented in this study are included in the article. Further inquiries can be directed to the corresponding author.
